# Kupffer-Cell-Targeted Carboxylesterase 1f Knockdown Deteriorates Lipopolysaccharide/D-Galactosamine-Induced Acute Liver Failure Through Regulating Cellular Polarization in Mice

**DOI:** 10.1155/cjgh/6410484

**Published:** 2024-12-21

**Authors:** Sai Zhao, Xue Yang, Yu He, Qian Yu, Liang-Ming Liu

**Affiliations:** ^1^Department of Infectious Diseases, Songjiang Hospital Affiliated to Shanghai Jiao Tong University School of Medicine, Shanghai, China; ^2^Department of Infectious Diseases, The Fourth Affiliated Hospital of Soochow University, Suzhou, China; ^3^Department of Infectious Diseases, Shanghai Songjiang Clinical Medical College of Nanjing Medical University, Shanghai, China

**Keywords:** acute liver failure, carboxylesterase 1f, gene knockdown, Kupffer cells, polarization phenotype

## Abstract

**Background:** Aims: Carboxylesterase (Ces)1f is implicated in protection against hepatic inflammation, but it is unclear whether the enzyme has an influence in polarization of Kupffer cells (KCs), the innate immune cells mediating hepatic inflammatory injury including acute liver failure (ALF). In the present study, we aim to explore KC polarization induced by Ces1f in mice with lipopolysaccharide/D-galactosamine (LPS/D-GalN)-induced ALF.

**Methods:** We adopted a novel delivery system, β-1,3-D-glucan-encapsulated Endoporter-siRNA particles, to specifically target KC Ces1f knockdown via tail vein injection in mice.

**Results:** Ces1f knockdown increased LPS/D-GalN-induced lethality as well as serum levels of alanine and aspartate transaminases, deteriorated hepatic inflammatory injury, and imbalanced hepatic oxidative stress molecules including myeloperoxidase, malondialdehyde, and superoxide dismutase in ALF. Ces1f knockdown also increased the levels of proinflammatory cytokines (tumor necrosis factor-*α* and interleukin-6) and decreased the levels of anti-inflammatory cytokine (interleukin-10) in LPS/D-Gal-induced ALF. Ces1f knockdown promoted KC M1 phenotype and marker expression (including CD86 and interleukin-1β), but inhibited M2 phenotype and marker expression (including CD163, CD206, and Arginase 1).

**Conclusions:** Our results suggest that Ces1f plays a hepatoprotective role through regulating KC polarization, which might contribute to anti-inflammatory and antioxidative effects in LPS/D-Gal-induced ALF mice.

## 1. Introduction

Acute liver failure (ALF) is a devastating clinical syndrome characterized by anorexia, ascites, coagulopathy, jaundice, and hepatic encephalopathy, resulting in multiorgan failure, immune paresis, high mortality, and poor prognosis [1]. The pathogenesis of ALF is systemic immune dysfunction contributing to irreversible massive hepatocyte death [2]. Liver-resident macrophages, Kupffer cells (KCs), reside within liver sinusoids and play a pivotal role in mediating the immune responses of liver inflammatory injuries [3], homeostasis, and tissue repair. Lipopolysaccharide (LPS) is a major chemical component embedded in the outer membrane of Gram-negative bacteria, also known as bacterial endotoxin, which activates the intrinsic immune system through toll-like receptor (TLR)4 and triggers a potent proinflammatory response. D-galactosamine (D-GalN) is also a well-known hepatotoxic substance that depletes the intracellular pool of uracil nucleotides in hepatocytes, amplifying the toxicity of LPS and exacerbating liver injury [4].

Macrophages can be activated by LPS, defined as classically activated or M1 polarization macrophages [5], characterized by CD86 expression as well as being involved in the secretion of proinflammatory factors, such as tumor necrosis factor (TNF), interleukin (IL)-1β, and IL-6 [5], initiating inflammatory response and mediating tissue damage. M1-polarized macrophages also release reactive oxygen species (ROS) (including inducible NO synthetase; iNOS), resulting in cytotoxic and inflammatory damage in liver tissues [5]. Under the influence of inflammation and oxidative stress, liver damage can further develop into liver failure [6]. In contrast, M2 macrophages are activated by IL-13 and IL-4, defined as alternatively activated or M2 polarization, resulting in the production of anti-inflammatory mediators, such as IL-10, arginase (Arg)1, CD163, and CD206 [7]. M2-polarized macrophages play fundamental roles in resolving inflammation and promoting tissue repairs [5]. Under some situations, macrophages have some plasticity and can switch from one polarization state to another [8]. In recent years, some scientists tried to use this characteristic of macrophages to treat immune inflammatory diseases, so as to reduce or repair tissue damage. However, due to the lack of understanding of the molecular mechanism regulating M1/M2 polarization, inflammatory macrophages (M1) encountered “metabolic roadblock” when transforming into repair phenotype [8].

It has been confirmed that the stimulation of injurious factors, including LPS, mainly causes disorder of lipid metabolism in hepatic macrophages, resulting in inhibition of reversible cholesterol transport and accumulation of intracellular cholesterol [9]. Abnormal cholesterol accumulation can enhance TLR4 signal transduction and inflammatory body activity, and induce cellular inflammatory release and innate immune response in macrophages [9]. The above metabolic processes of macrophages are mainly controlled by the nuclear transcription factor liver *X* receptor (LXR)*α* [10]. LXR*α* can affect the expression of a variety of enzymes and transporters and regulate homeostasis of cholesterol and fatty acid by forming heterodimers with retinoid *X* receptor (RXR) [11]. Previous reports have shown that LXR activity helps to protect mice from hepatic damage caused by toxic substances in ALF animal models [12, 13]. LXR*α* is activated by dephosphorylation of serine at positions 196 and 198 on macrophage proteins [14]. After dephosphorylation, LXR*α* reduces progression to hepatic inflammation induced by adipose deposition in steatohepatitis [15] and reduces M1 (while increases M2) macrophage infiltration in carbon tetrachloride (CCL4)-treated hepatic injury mice [16]. In vitro experiments have confirmed that LXR*α* activation can induce transformation from M1 to M2 polarization phenotype in LPS-stimulated KCs [16]. In order to find out further information about LXR*α* protection, Becares et al. [15] tested the damaged liver tissues by microarray and found that LXR*α* dephosphorylation promoted significant upregulation of Ces1f expression and decreased hepatic inflammatory injuries in mice [15]. This suggests that Ces1f plays an important role in LXR*α* protection of hepatic damage and KC inflammatory activity.

Ces1f is a member of the Ces1 family, and is a hydrolase that acts on esters and catalyzes hydrolysis of cholesterol esters and triglycerides (TGs) [17]. Ces1 has the highest concentration in the liver, and is involved in hepatic metabolism of drugs [18]. When devoid of Ces1 activity, prodrugs cannot be activated into active metabolites to exert their effect [18]. Hepatic Ces1 also has a role in regulating lipid and carbohydrate metabolism and lipid homeostasis [19]. Overexpression of hepatic Ces1 lowers hepatic TG and plasma glucose levels in diabetic mice [19]. However, Ces1 knockdown increases hepatic TG and plasma cholesterol levels [19]. In high-fat-diet-fed mice, Ces1 knockdown induces higher blood aspartate transaminase (AST) and alanine transaminase (ALT) levels, along with more severe hepatic histological injuries than in wild-type mice. Ces1f also exhibits significant hydrolase activities in cholesterol esters and TGs and controls hepatic lipid mobilization [17, 20]. However, there are few reports related to the roles of Ces1f in KC polarization and hepatic inflammatory responses. In this study, we used a mouse model of LPS/D-GalN-induced ALF to study the effects of Ces1f on KC polarization activity through KC-targeted Ces1f knockdown. Our results will help to clarify the role and mechanism of Ces1f in the pathogenesis of ALF.

## 2. Materials and Methods

### 2.1. Reagents

LPS and D-GalN were obtained from Sigma-Aldrich (St. Louis, MO, USA); EndoPorter was purchased from GeneTools LLC (Philomath, OR, USA); β-1,3-D-glucan was purchased from Thermo Scientific (Shanghai, China); siRNA synthesis was obtained from Shanghai Bioengineering Co. Ltd.; Trizol was purchased from Invitrogen (Shanghai, China); reverse transcription (RT) kit and SYBRGreen PCR kit were purchased from TaKaRa Bio Inc. (Tokyo, Japan); myeloperoxidase (MPO) enzyme assay kit, superoxide dismutase (SOD) assay kit, and malondialdehyde (MDA) assay kit were purchased from Nanjing Jiancheng Bioengineering Institute; mouse F4/80 antibody was purchased from ProteinTech Group (Wuhan, China); rabbit CD86 antibody was purchased from Cell Signaling Technology (Danvers, MA, USA); and rabbit CD163 antibody was purchased from Abcam (Cambridge, MA, USA).

### 2.2. siRNA and Preparation of β-1,3-D-Glucan-Encapsulated Endoporter-siRNA Particles

Mouse *Ces1f* gene sequences were deposited in GenBank (NM_144,930.2), and Ces1f-siRNA was designed according to the siRNA design principles on the Invitrogen website. The sequence with the best efficiency (gene location 1100) was used for the following experiments after the inhibitory efficiency studies (Table 1). A scrambled sequence was also designed for the experiment.

To load siRNA in glucan shells in vivo, β-1,3-D-glucan-encapsulated Endoporter-siRNA particles (GeRPs) were prepared as described previously [21]: 5 nmol siRNA was incubated with 50 nmol of Endopoter (Gene Tools) in 30 mM sodium acetate (pH 4.8) in a final volume of 20 μL for 15 min at room temperature. The siRNA-Endoporter solution was added to 1 mg (∼10^9^) glucan shells and vortex mixed and incubated for 1 h at 4°C. Tris/EDTA buffer (10 mM Tris and 1 mM EDTA, pH 7.4) was added to the particles to adjust the pH and incubated for 15 min at room temperature. The siRNA-loaded GeRPs were resuspended in PBS and vortex mixed to ensure homogeneity of the GeRP preparation. GeRPs were aliquoted into tubes for daily dosing and either flash-frozen in liquid nitrogen and stored at −20°C, or kept at 4°C. siRNA-Endoporter complexes were stable in GeRPs for at least 2 h at 37°C and 3 days at 4°C.

### 2.3. Animal Experiments

Male c57BL/6 mice (age 6–8 weeks, 20–25 g weight) were obtained from Jiesijie Laboratory Animal Co. Ltd. (Shanghai, China). The mice were bred in 12-h light/dark cycles at 25°C, and received standard rodent chow and water *ad libitum*. Animal experimental protocols were approved by the Animal Care and Use Committee of the Nanjing Medical University and conformed to the Guide for the Care and Use of Laboratory Animals (IACUC-2106040).

**Figure 1:**
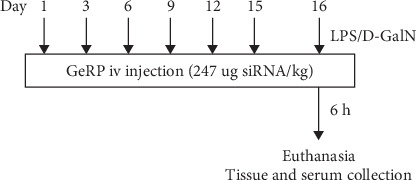
Injection times.

A total of 30 mice were divided into five groups randomly. Acute liver injury was induced by intraperitoneal injection of 50 μg/kg LPS plus 800 mg/kg D-GalN [22–24] (LPS/D-GalN). Before challenge, mice were pretreated with Ces1f-siRNA-GeRPs (1.5 mg GeRPs/kg) six times via tail vein injection according to the time points within 15 days [25] (Figure 1) (Ces1f-GeRPs + LPS/D-GalN). The control mice received intraperitoneal injections of an equal quantity of normal saline after pretreatment with Ces1f-GeRPs or scrambled GeRPs (SCR-GeRPs) or PBS (Control) via tail vein injection six times in 15 days. On day 16, mice were challenged with LPS/D-GalN. After 6 h, mice were anesthetized, and livers and serum were collected. Another 50 mice were analyzed for survival rates (10 mice/group).

### 2.4. Biochemical Assays

Sera were separated from the whole blood after centrifugation. Serum AST and ALT activities were measured using an automatic biochemistry analyzer (Sysmex, Kobe, Japan). The content of liver MPO, SOD, and MDA was detected using assay kits.

### 2.5. Fluorescence In Situ Hybridization (FISH)

FISH probe of Ces1f was designed and synthesized by UBI (Shanghai, China). The probe sequence was as follows: 5′ DIG-TGGAGCACCGAAGACAGAGTAGACATCA-DIG 3′. Liver sections were fixed (4% paraformaldehyde), dehydrated in sucrose solution, and then digested with proteinase K (20 μg/mL, 37°C for 10 min). After hybridization overnight at 37°C with the Ces1f probe, the digoxigenin antibody was used to visualize the positive hybridization signals. Hepatic FISH was performed using fluorescence microscopy (Nikon, Tokyo, Japan). Ces1f mRNA expression was quantified by counting the fluorescence intensity of positive hepatic cells in high-power fluorescence microscope fields (630×). Five sections were visualized per group, and at least three fields were randomly selected to calculate the fluorescence intensity per section. The levels of Ces1f expression were standardized with the fluorescence intensity per cell in the sections.

### 2.6. Enzyme-Linked Immunosorbent Assay

Serum TNF-*α*, IL-6, and IL-10 (eBioscience, San Diego, CA, USA) levels were determined by ELISA.

### 2.7. Immunofluorescence Staining

For immunofluorescence staining, liver sections were dewaxed in xylene and rehydrated in graded alcohols. Following dehydration and antigen retrieval, tissue sections were blocked at 25°C for 1 h and then incubated with the following primary antibodies overnight: F4/80 (1:100, ProteinTech Group), Ces1f (1:100, polyclonal antibody produced by Abmart, Shanghai), CD86 (1:200, Cell Signaling Technology), and CD163 (1:100, Abcam). Sections were incubated at 37°C for 1 h with the following secondary antibodies: fluorescein isothiocyanate (1:100); cyanine 3 (1:100)-conjugated antirabbit or antimouse IgG (Beyotime). The nuclei were stained with 4′,6-diamidino-2-phenylindole (DAPI) (Beyotime). Images were captured with an inverted fluorescence microscope (Olympus Corporation, Tokyo, Japan). Six slides were observed in each group, each specimen was observed in three random visual fields, and the average number of double-positive cells in each specimen was calculated. The F4/80 positive cells (F4/80+), which represent the KCs, were calculated in the same way.

### 2.8. Terminal Deoxynucleotidyl Transferase dUTP Nick End Labeling (TUNEL) Assay

The terminal deoxynucleotidyl TUNEL assay kit (Roche, Basel, Switzerland) was used to determine the presence of apoptotic cells. Tissue sections were dewaxed and rehydrated, and incubated with proteinase K working solution and rinsed with PBS. Slides were incubated with permeabilization solution and rinsed again with PBS. TUNEL reaction mixture was added. The slides were incubated for 1 h in a humidified atmosphere in the dark and rinsed with PBS. After adding 50 μL Converter-POD, slides were again incubated and washed, followed by the addition of 100 μL diaminobenzidine substrate, incubation, and rinsing with PBS. DAPI staining made all hepatocyte nuclei show blue fluorescence, and TUNEL staining made apoptotic cells show green fluorescence. The TUNEL‐positive cells were examined through fluorescence microscopy (Olympus). Three fields were randomly selected for each slide to calculate the percentage of apoptotic cells to the total number of cells.

### 2.9. Western Blotting

Liver tissues were collected and lysed with RIPA lysis buffer (Beyotime) on ice. Following centrifugation at 12,000 r/min, 4°C for 15 min, the protein was collected and used for concentration measurement with a BCA kit (Beyotime). Total protein (20 μg) from each sample was separated by SDS-PAGE (10%) and transferred electrophoretically to nitrocellulose filter membrane (Beyotime). The membranes were blocked with 5% nonfat milk in Tris-buffered saline containing 0.5% Tween-20 and incubated overnight at 4°C with the following primary antibodies: Ces1f (1:800) and β-actin (1:2000). The membranes were incubated with HRP-conjugated antimouse (1:5000) and antirabbit IgG (1:5000) (Beyotime) for 2 h at room temperature. The protein bands were developed using ECL luminescence chromogenic reagent (Millipore, Burlington, MA, USA) and quantified using Bio-Rad Quantity one version 4.6.7 software.

### 2.10. Reverse Transcription Quantitative Polymerase Chain Reaction Analysis

Total mRNA was extracted from the liver tissues using Trizol (Thermo Scientific). RT was performed following the protocol of the PrimeScript RT reagent kit with gDNA eraser (TaKaRa, Tokyo, Japan). The Permix Taq kit (TaKaRa) was used for quantitative polymerase chain reaction (qPCR). All of the reactions were performed using the cFX96 Touch Real-Time PCR detection system (Bio-Rad, Hercules, CA, USA). GAPDH was amplified as an internal control, and the relative mRNA expression levels were calculated using the 2^−ΔΔCt^ method. The used primers are shown in Table 2.

### 2.11. Statistical Analysis

Statistical analysis was performed using GraphPad Prism software version 8.0. All data are presented as the mean ± standard deviation (SD). Comparison between multiple groups was evaluated for significance using one-way analysis of variance followed by Tukey's *post hoc* analysis. The survival rates were determined using log-rank test. *p* < 0.05 was deemed statistically significant.

## 3. Results

### 3.1. Hepatic Ces1f Expression in siRNA-Pretreated ALF Mice

**Figure 2:**
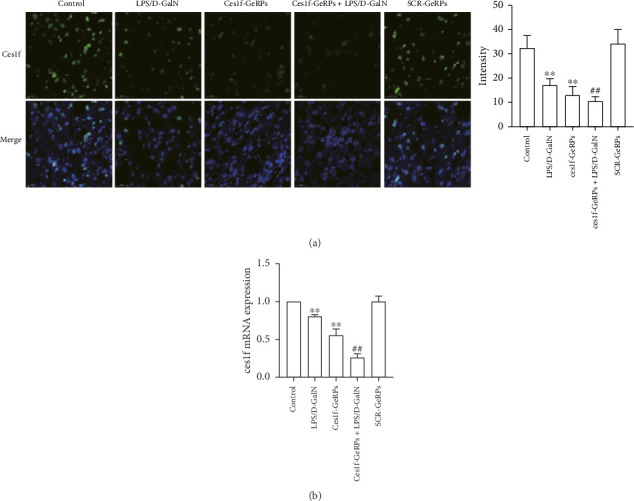
Hepatic Ces1f expression in LPS/D-GalN-challenged mice with KC-targeted gene knockdown. Mice were challenged by LPS/D-GalN injection intraperitoneally after KC-targeted Ces1f knockdown using tail vein administration of GeRPs. (a) Ces1f mRNA expression in liver tissues with FISH assay. Left panel shows a representative picture of hepatocellular Ces1f mRNA expression under fluorescence microscopy, and right panel shows a relative fluorescence intensity of Ces1f mRNA in liver after normalization to cell numbers. Data represent means ± SD (*n* = 6 each group). Nuclei were stained blue with DAPI, and cytoplasmic Ces1f was stained green (×630, scale bar 20 μm). (b) Ces1f mRNA expression in liver tissues using RT-qPCR. Relative expression levels of Ces1f mRNA were detected in liver after normalization to control through RT-qPCR. Data represent means ± SD (*n* = 6 each group). ⁣^∗^*p* < 0.05 and ⁣^∗∗^*p* < 0.01 versus control mice [Ces1f-siRNA(−)LPS/D-GalN(−)]; ^#^*p* < 0.05 and ^##^*p* < 0.01 versus LPS/D-GalN-challenged mice [Ces1f-siRNA(−)LPS/D-GalN(+)].

GeRPs delivery system can effectively and specifically target phagocytes to mediate gene silencing in intact mice [21, 25]. In this experiment, we tested hepatic Ces1f mRNA expression using real-time PCR and FISH methods, and Ces1f protein expression using western blotting. Hepatic levels of Ces1f mRNA and protein were downregulated in both LPS/D-GalN-challenged and Ces1f knockdown mice (both *p* < 0.01) and had a further decline in Ces1f-siRNA-pretreated ALF mice (*p* < 0.01) (Figure 2).

### 3.2. KC Ces1f Expression in siRNA-Pretreated ALF Mice

**Figure 3:**
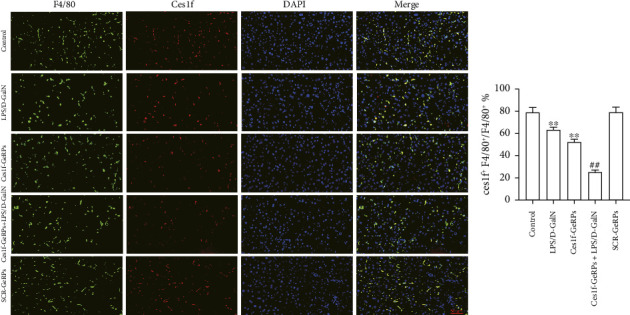
KC Ces1f expression in LPS/D-GalN-challenged mice with KC-targeted gene knockdown. Mice were challenged by LPS/D-GalN injection intraperitoneally after KC-targeted Ces1f knockdown using tail vein administration of GeRPs. Double immunofluorescence staining was conducted to assess Ces1f expression in KCs. Left panel shows representative pictures of KC Ces1f expression (F4/80^+^Ces1f^+^) under fluorescence microscopy, and right panel shows the percentages of F4/80^+^Ces1f^+^ cells. Data represent means ± SD (*n* = 6 each group). Nuclei were stained blue with DAPI, KC marker F4/80 was stained green, and cytoplasmic Ces1f was stained red. ⁣^∗^*p* < 0.05 and ⁣^∗∗^*p* < 0.01 versus control mice [Ces1f-siRNA(−)LPS/D-GalN(−)]; ^#^*p* < 0.05 and ^##^*p* < 0.01 versus LPS/D-GalN-challenged mice [Ces1f-siRNA(−)LPS/D-GalN(+)].

We exploited double immunofluorescence staining to assay the levels of KC Ces1f in LPS/D-GalN-induced ALF mice after KC-targeted gene knockdown via GeRPs delivery. The mean value of four randomly selected areas was used to calculate the proportion of coexpression or colocation of KCs (F4/80^+^, green) and Ces1f-positive cells (red) [Ces1f^+^F4/80^+^/F4/80^+^ (%)]. As illustrated in Figure 3, both LPS/D-GalN-challenged and Ces1f-GeRPs mice had a decrease in the percentages of F4/80^+^Ces1f^+^ cells compared with controls (both *p* < 0.01). Following pretreatment with Ces1f-GeRPs, double-positive KCs decreased more in LPS/D-GalN-challenged mice than in unprocessed pairs (*p* < 0.01).

### 3.3. KC Ces1f Knockdown Aggravated LPS/D-GalN-Induced ALF in Mice

**Figure 4:**
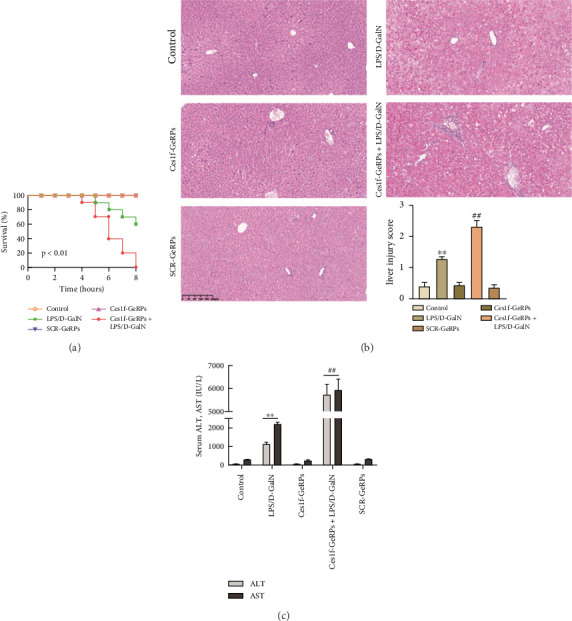
Effects of KC Ces1f knockdown on animal survival, hepatic injury, and blood ALT/AST levels in ALF mice. Mice were challenged by LPS/D-GalN injection intraperitoneally after KC-targeted Ces1f knockdown using tail vein administration of GeRPs. (a) Survival. Mouse survival rates in five groups at different times within 8 h after challenge with LPS/D-GalN (*n* = 10 each group). (b) Morphological appearance. A representative hepatic staining using hematoxylin–eosin (H&E) is shown, and liver injury scores are shown in the bottom right corner. Data represent means ± SD (*n* = 6 each group). (c) Serum ALT and AST levels. Data represent means ± SD (*n* = 6 each group). ⁣^∗^*p* < 0.05 and ⁣^∗∗^*p* < 0.01 versus control mice [Ces1f-siRNA(−)LPS/D-GalN(−)]; ^#^*p* < 0.05 and ^##^*p* < 0.01 versus LPS/D-GalN-challenged mice [Ces1f-siRNA(−)LPS/D-GalN(+)].

To investigate the role of Ces1f in ALF, mice were challenged with LPS/D-GalN to assay survival rates, liver histopathology, and blood ALT and AST. LPS/D-GalN challenge caused 60% lethality within 8 h after injection, whereas the mortality increased to 100% after Ces1f-GeRP pretreatment (*p* < 0.01) (Figure 4(a)). LPS/D-GalN-challenged mice also showed characteristically disordered hepatic architecture, pronounced lobular edema, sinusoidal congestion, heavy centrilobular ballooning, and high histological scores, and Ces1f-siRNA pretreatment exacerbated liver damage and histological score, accompanied by extensive hepatocellular necrosis (Figure 4(b)). Serum ALT and AST levels were elevated in LPS/D-GalN-challenged mice, and Ces1f-GeRP pretreatment further increased the levels of serum aminotransferases (*p* < 0.01) (Figure 4(c)). These results suggest that KC Ces1f may help protect against LPS/D-GalN-induced ALF in mice.

### 3.4. KC Ces1f-Knockdown Worsen Liver Oxidative Stress in LPS/D-GalN-Induced ALF Mice

**Figure 5:**
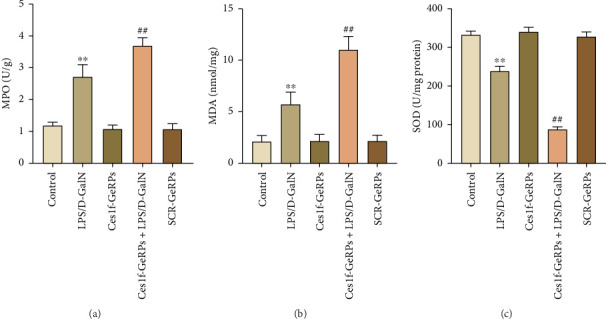
Activities of hepatic MPO, MDA, and SOD in LPS/D-GalN-challenged mice with KC-targeted gene knockdown. Mice were challenged by LPS/D-GalN injection intraperitoneally after KC-targeted Ces1f knockdown using tail vein administration of GeRPs. The levels of MPO, MDA, and SOD were evaluated by specific commercial assay kits. (a) MPO. (b) MDA. (c) SOD. Data represent means ± SD (*n* = 6 each group). ⁣^∗^*p* < 0.05 and ⁣^∗∗^*p* < 0.01 versus control mice [Ces1f-siRNA(−)LPS/D-GalN(−)]; ^#^*p* < 0.05 and ^##^*p* < 0.01 versus LPS/D-GalN-challenged mice [Ces1f-siRNA(−)LPS/D-GalN(+)].

Oxidative stress plays an important role in the occurrence and development of acute liver injury or failure. There are a variety of molecules associated with oxidative stress. Among them, MPO is a surface indicator of neutrophils and a surrogate for determining the degree of neutrophilic activation [26]; MDA is the most prevalent biomarker of lipid peroxidation during liver injury [27, 28]; and SOD is the major antioxidant enzyme in the liver [29]. SOD is a key enzyme that catalyzes the dismutation of superoxide radicals resulting from cellular oxidative metabolism into hydrogen peroxide, and prevents LPS-induced penetration [27]. To investigate the effects of KC Ces1f on oxidative stress in acute liver injury, we determined the activities of MDA, MPO, and SOD by gene knockdown. There was a substantial increase in MPO and MDA activities (both *p* < 0.01) and a significant decrease in SOD content (*p* < 0.01) in liver tissues in LPS/D-GalN-induced ALF mice. The abnormity was more pronounced following Ces1f-siRNA pretreatment (Figure 5). This suggests that KC Ces1f ameliorates hepatic oxidative stress injury in ALF mice.

### 3.5. KC Ces1f-Knockdown Exacerbated Hepatic Apoptosis in LPS/D-GalN-Induced ALF Mice

**Figure 6:**
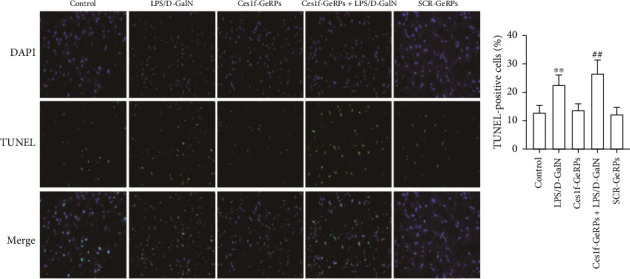
Effects of KC Ces1f knockdown on hepatic apoptosis in ALF mice. Mice were challenged by LPS/D-GalN injection intraperitoneally after KC-targeted Ces1f knockdown using tail vein administration of GeRPs. Hepatic apoptosis was analyzed by TUNEL staining. The TUNEL‐positive cells were examined through fluorescence microscopy. DAPI staining made all hepatocyte nuclei show blue fluorescence, and TUNEL staining made apoptotic cells show green fluorescence. Representative figures of hepatic TUNEL staining are shown, and the proportion of TUNEL-positive cells is shown on the right. Data represent means ± SD (*n* = 6 each group). ⁣^∗^*p* < 0.05 and ⁣^∗∗^*p* < 0.01 versus control mice [Ces1f-siRNA(−)LPS/D-GalN(−)]; ^#^*p* < 0.05 and ^##^*p* < 0.01 versus LPS/D-GalN-challenged mice [Ces1f-siRNA(−)LPS/D-GalN(+)].

The apoptotic cells in liver tissues were analyzed by TUNEL staining. TUNEL-positive cells increased significantly in mouse liver tissues after LPS/D-GalN administration (*p* < 0.01) and became worse following Ces1f-siRNA pretreatment (*p* < 0.01) in ALF mice (Figure 6). This suggests that Ces1f alleviates LPS/D-GalN-induced hepatic apoptosis in mice.

### 3.6. KC Ces1f-Knockdown Exacerbated Cytokine Disorder in LPS/D-GalN-Induced ALF Mice

**Figure 7:**
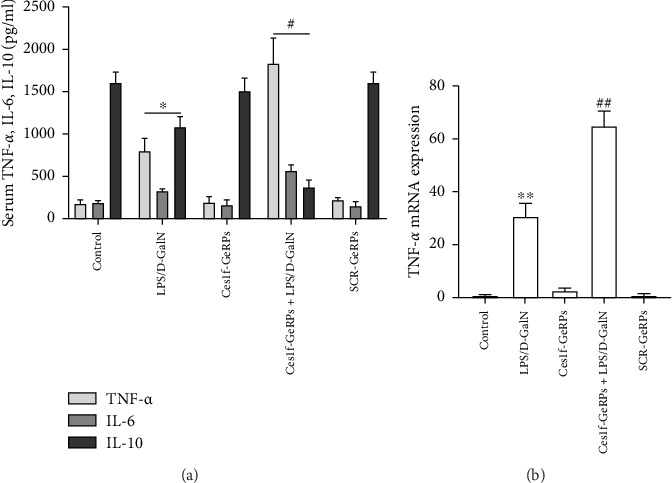
Effects of KC Ces1f knockdown on secretion of TNF-*α*, IL-6, and IL-10 in ALF mice. Mice were challenged by LPS/D-GalN injection intraperitoneally after KC-targeted Ces1f knockdown using tail vein administration of GeRPs. (a) Serum cytokines were analyzed by ELISA. (b) mRNA expression of TNF-*α* in liver tissues using RT-qPCR. Data represent means ± SD (*n* = 6 each group). ⁣^∗^*p* < 0.05 and ⁣^∗∗^*p* < 0.01 versus control mice [Ces1f-siRNA(−)LPS/D-GalN(−)]; ^#^*p* < 0.05 and ^##^*p* < 0.01 versus LPS/D-GalN-challenged mice [Ces1f-siRNA(−)LPS/D-GalN(+)].

To study the influence of Ces1f on hepatic inflammation, we measured a panel of cytokines including proinflammatory cytokines TNF-*α* and IL-6, and anti-inflammatory cytokine IL-10 in blood. As shown in Figure 7(a), there were significant increases of serum TNF-*α* and IL-6 (both *p* < 0.01), a significant decrease of IL-10 (*p* < 0.01) in LPS/D-GalN-challenged mice. Pretreatment with Ces1f-siRNA exacerbated the above disorder. In addition, we detected the mRNA levels of TNF-*α* in liver tissues. In line with serum TNF-*α*, hepatic TNF-*α* mRNA had a similar change tendency among the experimental groups (Figure 7(b)). This suggests that Ces1f reduces inflammatory releases in ALF mice.

### 3.7. KC Ces1f-Knockdown Boosted KC M1 Polarization in LPS/D-GalN-Induced ALF Mice

**Figure 8:**
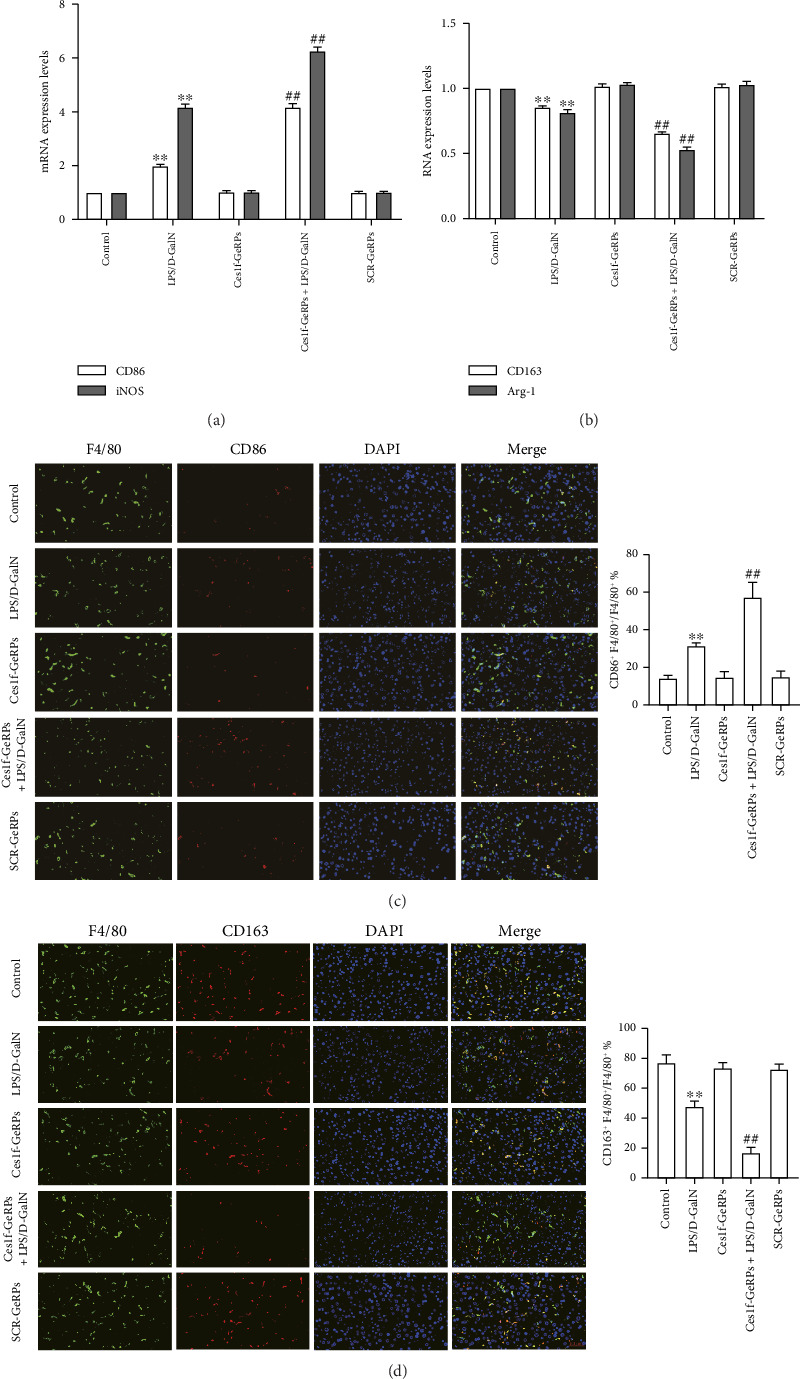
Effects of Ces1f knockdown on KC-polarized activities in LPS/D-GalN-induced ALF. Mice were challenged by LPS/D-GalN injection intraperitoneally after KC-targeted Ces1f knockdown using tail vein administration of GeRPs. mRNA expression was assayed by RT-qPCR, and polarized KCs were evaluated through double immunofluorescence staining. Nuclei were stained blue with DAPI, KCs stained green with F4/80, and polarized cells stained red with biomarkers CD86/CD163. M1 KCs were F4/80^+^CD86^+^ cells, and M2 KCs were F4/80^+^CD163^+^ cells. (a) mRNA expression of M1 biomarkers (CD86 and iNOS) in liver tissues. (b) mRNA expression of M2 biomarkers (CD163 and Arg-1) in liver tissues. (c) Representative pictures of M1 phenotype KCs are shown, and right panel shows the percentages of M1-polarized cells to total KCs [(CD86^+^F4/80^+^/F4/80^+^)%]. (d) Representative pictures of M2 phenotype KCs are shown, and right panel shows the percentages of M2-polarized cells to total KCs [(CD163^+^F4/80^+^/F4/80^+^)%]. Data represent means ± SD (*n* = 6 each group). ⁣^∗^*p* < 0.05 and ⁣^∗∗^*p* < 0.01 versus control mice [Ces1f-siRNA(−)LPS/D-GalN(−)]; ^#^*p* < 0.05 and ^##^*p* < 0.01 versus LPS/D-GalN-challenged mice [Ces1f-siRNA(−)LPS/D-GalN(+)].

To investigate the effects of Ces1f knockdown on KC polarization in LPS/D-GalN-induced ALF, we tested the levels of KC M1 and M2 polarization biomarkers in liver tissues. CD86 and IL-1β are two classic polarization biomarkers of M1 KCs or macrophages, whereas CD206 and Arg-1 are the polarization biomarkers of M2 cells. Real-time PCR showed that CD86 and IL-1β mRNA increased in LPS/D-GalN-induced ALF mice and had higher levels following Ces1f-siRNA pretreatment (both *p* < 0.01) (Figure 8(a)). Conversely, CD206 and Arg-1 mRNA decreased in ALF mice (both *p* < 0.01) and had lower levels following Ces1f-siRNA pretreatment (both *p* < 0.01) (Figure 8(b)). We used immunofluorescence double staining to label F4/80, a specific cell surface marker of KCs, and CD86 (M1 marker) and CD163 (M2 marker) to determine KC polarization phenotype. The percentages of F4/80^+^CD86^+^ KCs increased in LPS/D-GalN-induced ALF mice (*p* < 0.01) and had higher levels following Ces1f-siRNA pretreatment with GeRPs (*p* < 0.01) (Figure 8(c)). Conversely, the percentages of F4/80^+^CD163^+^ KCs decreased in LPS/D-GalN-induced ALF mice (*p* < 0.01) and further declined after Ces1f-siRNA pretreatment (*p* < 0.01) (Figure 8(d)). These results indicate that KC-targeted Ces1f knockdown promotes LPS/D-GalN-induced M1/M2 polarization imbalance. Ces1f may help to maintain KC repair and reduce KC inflammatory polarization phenotype.

## 4. Discussion

Ces1f is a member of the Ces1 family of carboxylesterases. Ces1 is the most abundant in liver and macrophages and mainly exists in the microsomes of the endoplasmic reticulum. The gene encoding *Ces1* is located on chromosome 16 and contains 14 exons. Ces1 can metabolize chemicals containing ester bonds and amide bonds. It is an important phase I drug-metabolizing enzyme in the body and can metabolize many classes of drugs [30–32]. Under physiological conditions, the important function of Ces1 is to regulate the metabolic balance of the liver. Previous studies have confirmed that Ces1 can promote TG, cholesterol, and carbohydrate metabolism in the liver, accelerate lipidolysis and fatty acid oxidation, and lower glycemia [19, 33]. Insufficient Ces1 activity can cause liver metabolic disorder and liver injury [34]. So far, there are few studies on Ces1f. Some reports show that Ces1f also has hydrolase activity [20] and perhaps also deacetylase activity [35]. The regulatory effects of Ces1f on hepatic inflammatory responses are an important discovery in hepatology research in recent years [15].

In order to further analyze the roles and mechanisms of Ces1f in the hepatic inflammatory injury in ALF, we used in vivo technology of KC-targeted gene knockdown—the method of GeRP injection [21]. This technology mainly uses GeRPs to knock down the target gene through tail vein injection six times in 15 days. β-1,3-D-glucan can be specifically recognized by Dectin-1 receptor on the surface of KC, and GeRPs can be taken up and endocytosed into KCs. Under the action of Endoporter, phagocytic vesicles release siRNA to achieve KC-targeted gene knockdown. We found that expression of hepatic Ces1f mRNA and protein was significantly reduced after injection of interferential GeRPs (GeRPs-Ces1f-siRNA). To analyze the relationship between hepatic Ces1f downregulation and in vivo KC gene knockdown, we used double fluorescence dye-labeling technology in liver tissues. Among them, F4/80, a common surface marker of KCs, showed green fluorescence after staining, and Ces1f showed red fluorescence. The injection of GeRPs-Ces1f-siRNA caused a significant decrease of double-positive cells (F4/80^+^ces1f^+^ KCs). This suggests that GeRP injection technology can effectively knock down the expression of KC Ces1f in vivo.

Subsequently, we analyzed the effects of KC-targeted Ces1f knockdown on hepatic inflammatory injury of ALF in vivo. We found that, in LPS/D-GalN-induced ALF mice, the intrahepatic damage inflammation and oxidative stress responses were significantly activated, while the expression of liver (KC) Ces1f was significantly downregulated. After the injection of GeRPs-ces1f-siRNA, liver (KC) Ces1f expression was further downregulated in ALF mice; that is, the downregulation of Ces1f expression was superimposed in the liver (mainly KC) in ALF mice with KC-targeted gene knockdown. With the further downregulation of Ces1f in KCs (including liver), mouse mortality increased, and hepatic inflammatory injury and oxidative stress reaction were significantly aggravated in ALF mice injected with GeRPs-ces1f-siRNA. This suggests that Ces1f inhibits hepatic inflammatory damage.

To further understand the roles of Ces1f in the inflammatory responses of liver tissues, we analyzed the effect of Ces1f on KC-polarized activity in vivo. Hepatic inflammatory response is closely related to the phenotypic state of KC polarization. Among them, M1 is LPS and/or interferon *γ* stimulation-induced polarized KC, mainly releasing proinflammatory enzyme molecules and cytokines such as CD86, iNOS, TNF-*α*, and IL-1β [36, 37]. These factors or molecules can cause liver immune inflammation and ALF [38]; in contrast, M2-polarized cells are mainly induced by IL-4 and/or IL-13, and can express and release anti-inflammatory enzyme molecules and cytokines such as CD206, CD163, IL-10, Arg-1, scavenging receptor, and mannose receptor, which are related to tissue repair, remodeling, and immunoregulation [36, 37]. Our study found that in normal liver tissues, M1-polarized markers CD86 and IL-1β/M2-polarized markers CD206 and Arg-1 mRNA were significantly expressed, indicating that hepatic M1/M2-polarized cells are in a relatively balanced state. After LPS/D-GalN challenge, the levels of hepatic M1/M2 markers were significantly imbalanced, and the M1 markers were upregulated, while the M2 markers were downregulated. Following pre-knockdown of KC Ces1f, we found that the M1 markers were upregulated and M2 markers were downregulated more significantly in ALF. Subsequently, we further analyzed the numbers of M1/M2 phenotype KCs by double immunofluorescence staining. The common marker of KCs is still F4/80, which shows green fluorescence after staining, while both M1 marker protein CD86 and M2 marker protein CD163 show red fluorescence. The results were similar to the above gene expression imbalance. In LPS/D-GalN-induced ALF, M1 phenotype KCs were significantly increased, while M2 phenotype KCs were significantly decreased in liver tissues. After knockdown of KC Ces1f, the imbalance of increased M1/decreased M2 cells was more serious; at this time, the degrees of hepatic inflammatory injury and oxidative stress were also significantly aggravated. This indicates that in LPS/D-GalN-induced ALF, anti-inflammatory or reparative M2 cells have a tendency to transform into inflammatory M1 cells, and Ces1f knockdown aggravates the imbalance and transformation trend of KC-polarized phenotypes.

Based on the above studies, we have the following three conclusions: (1) KC (or liver) Ces1f expression is downregulated in ALF; (2) KC-targeted knockdown of Ces1f gene can aggravate hepatic inflammatory injury in ALF; and (3) imbalance of KC polarization is further aggravated in ALF after KC-targeted knockdown of Ces1f gene. Therefore, we conclude that Ces1f may be a protective factor of hepatic inflammatory injury, and its inhibitory effects on liver inflammation may result from the maintenance of KC polarization balance. Such a study helps to clarify the substance metabolism mechanism of inflammatory responses in the process of ALF, which may provide a new way to treat ALF through targeting substance metabolism enzyme molecules in the future.

## Figures and Tables

**Table 1:** List of siRNA sequences used in the study.

siRNA	Sequences (5′ ⟶ 3′)	
Scrambled (SCR)	Sense	UUCUCCGAACGUGUCACGUTT
	Antisense	ACGUGACACGUUCGGAGAATT

Ces1f	Sense	GCUGGCUUCUGCCAACAAUTT
	Antisense	AUUGUUGGCAGAAGCCAGCTT

**Table 2:** Primers used to detect gene mRNA expression by q-PCR.

Gene	Forward	Reverse
GAPDH	5′-GACATGCCGCCTGGAGAAAC-3′	5′-GTCCACCACCCTGTTGCTGTAG-3′

Ces1f	5′-TTTGCACTGCCTACCAGAGTC-3′	5′-GGTGGTGAAGAGGGGTTTCC-3′

CD86	5′-TCCAAGTTTTTGGGCAATGTC-3′	5′-CCTATGAGTGTGCACTGAGTTAAACA-3′

iNOS	5′-AATCTTGGAGCGAGTTGTGG-3′	5′-CAGGAAGTAGGTGAGGGCTTG-3′

CD163	5′-TGGGTGGGGAAAGCATAACT-3′	5′-AAGTTGTCGTCACACACCGT-3′

Arg1 TNF-*α*	5′-CTCCAAGCCAAAGTCCTTAGAG-3′ 5′-GCGACGTGGAACTGGCAGAAG-3′	5′-AGGAGCTGTCATTAGGGACATC-3′ 5′-GTGGTTTGTGAGTGTGAGGGTCTG-3′

## Data Availability

The data that support the findings of this study are available from the corresponding author upon reasonable request.
